# Frequency Response and Gap Tuning for Nonlinear Electrical Oscillator Networks

**DOI:** 10.1371/journal.pone.0078009

**Published:** 2013-11-04

**Authors:** Harish S. Bhat, Garnet J. Vaz

**Affiliations:** Applied Mathematics Unit, University of California Merced, Merced, California, United States of America; Universidad de Zarazoga, Spain

## Abstract

We study nonlinear electrical oscillator networks, the smallest example of which consists of a voltage-dependent capacitor, an inductor, and a resistor driven by a pure tone source. By allowing the network topology to be that of any connected graph, such circuits generalize spatially discrete nonlinear transmission lines/lattices that have proven useful in high-frequency analog devices. For such networks, we develop two algorithms to compute the steady-state response when a subset of nodes are driven at the same fixed frequency. The algorithms we devise are orders of magnitude more accurate and efficient than stepping towards the steady-state using a standard numerical integrator. We seek to enhance a given network's nonlinear behavior by altering the eigenvalues of the graph Laplacian, i.e., the resonances of the linearized system. We develop a Newton-type method that solves for the network inductances such that the graph Laplacian achieves a desired set of eigenvalues; this method enables one to move the eigenvalues while keeping the network topology fixed. Running numerical experiments using three different random graph models, we show that shrinking the gap between the graph Laplacian's first two eigenvalues dramatically improves a network's ability to (i) transfer energy to higher harmonics, and (ii) generate large-amplitude signals. Our results shed light on the relationship between a network's structure, encoded by the graph Laplacian, and its function, defined in this case by the presence of strongly nonlinear effects in the frequency response.

## Introduction

**Figure 1:**
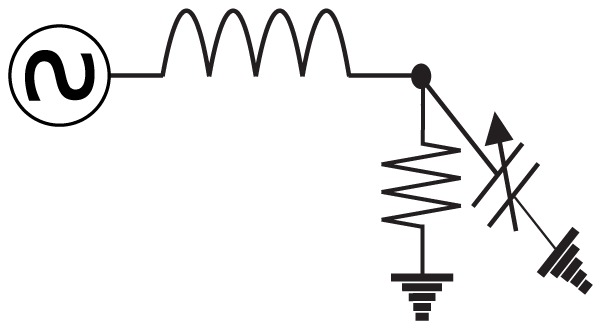
Schematic of a single nonlinear oscillator. This oscillator is the basic building block of the networks considered in this paper. The circuit contains one inductor, one voltage-dependent capacitor, one source, and one resistor.

Networks of nonlinear electrical oscillators have found recent application in several microwave frequency analog devices [1]–[6]. The fundamental unit in these networks is a nonlinear oscillator wired as in Figure 1; this oscillator consists of one inductor, one voltage-dependent capacitor, one source, and one sink (a resistor). While many nonlinear oscillatory circuits have been studied for their chaotic behavior, the particular oscillator in Figure 1 does not exhibit sensitive dependence on initial conditions in the regime of operation that we consider. Instead, assuming the source is of the form 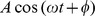, the oscillator reaches a steady-state consisting of a sum of harmonics with fundamental frequency 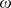 [7].

**Figure 2:**
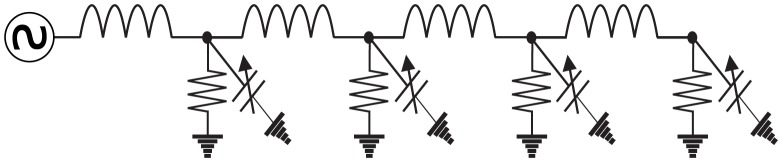
An example of a nonlinear transmission line. A nonlinear transmission line is a nonlinear electrical network on a one-dimensional linear graph.

**Figure 3:**
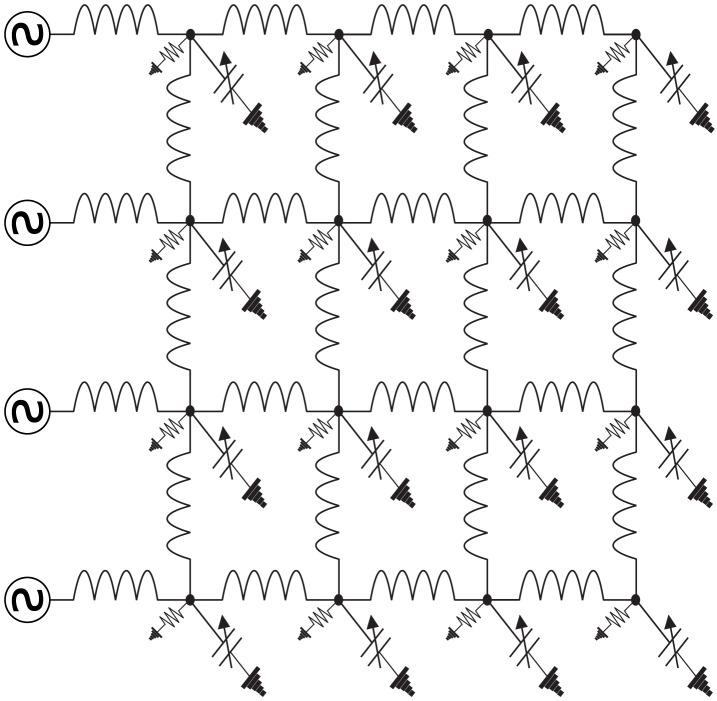
An example of a nonlinear lattice. A nonlinear lattice is a nonlinear electrical network on a two-dimensional rectangular grid graph.

When networks of these oscillators have been studied, the network topology has either been a one-dimensional linear chain, in which case the circuit is called a nonlinear transmission line [8]–[13]—see Figure 2, or a two-dimensional rectangular lattice [14]–[18]—see Figure 3. Even if each individual block in the chain/lattice is weakly nonlinear, the overall circuit can exhibit strongly nonlinear behavior. It is this property that is exploited for microwave device applications, enabling low-frequency, low-power inputs to be transformed into high-frequency, high-power outputs.

The first objective of this work is to develop numerical algorithms to compute the frequency response of a nonlinear electrical network with topology given by an arbitrary connected graph. Here we are motivated by the successful application of computational techniques in the design of the high-frequency analog devices referenced above. As we show, to compute steady-state solutions with comparable accuracy, both the perturbative and iterative algorithms developed in this paper require orders of magnitude less computational time than standard numerical integration. While the perturbative algorithm generalizes derivations given in prior work [7], [18], the iterative algorithm has not been previously applied to nonlinear electrical networks. Both new algorithms show exponential convergence in the number of iterations, and for a test problem on a network with 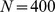 nodes, less than 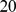 iterations are required to achieve machine precision errors.

The second objective of this work is to relate structural properties of the network to the dynamics of the nonlinear oscillator system. The derivation of the perturbative algorithm indicates that nonlinearity in the electrical network manifests itself through energy transfer from the fundamental forcing frequency to higher harmonics. This helps us understand why properties such as amplitude boosting [7], [18] and frequency upconversion [1], observed in nonlinear electrical networks with regular lattice topologies, can be expected when the topology is that of a random, disordered network. Additionally, we observe that an inductance-weighted graph Laplacian matrix features prominently in both algorithms for computing the steady-state solution. This graph Laplacian matrix encodes the structure of the network, and its eigenvalues are the squares of the resonant frequencies for the undamped, linear version of the circuit. Driving the damped, linearized circuit at one of these resonances results in large amplitude outputs. It is reasonable to hypothesize that the locations of these resonances play a large role in the dynamics of the nonlinear network.

This motivates the following question: how do the eigenvalues of the graph Laplacian influence the nonlinear network's properties of frequency upconversion and amplitude boosting? While it is possible to alter the spectrum of the graph Laplacian by changing the node-edge relationships in the graph, we can also change its spectrum by keeping the topology fixed and manipulating the network's inductances. We formulate and solve the inverse problem of finding the inductances such that the graph Laplacian achieves a prescribed spectrum. The solution proceeds via a Newton-type algorithm that takes the desired spectrum as input and iteratively alters the inductances until a convergence criterion is met.

For three types of random graphs, we find that the Newton-type method effectively finds circuit inductances that close the gap between the first two eigenvalues of the graph Laplacian. We conduct a series of numerical experiments to examine the effect of closing this eigenvalue gap on a given circuit's ability (i) to transfer energy from the fundamental driving frequency to higher harmonics, and (ii) to generate high-amplitude output signals. The results indicate that the two metrics (i-ii) can be improved dramatically by closing the gap between the graph Laplacian's first two eigenvalues. Table 1 shows results we obtained for graphs with 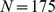 nodes. Though this a small portion of the results we describe later, this table already illustrates the effect of gap tuning on network performance. Note that each *pre* and *post* circuit have the same graph topology, differing only in their edge inductances.

**Table 1:** Portion of results for graphs with 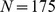 nodes.

	% of energy in higher harmonics		Maximum magnitude voltage	
	Pre	Post	Pre	Post
Barabási-Albert (BA)	0.410	4.063	0.01548	0.31684
Watts-Strogatz (WS)	1.006	8.701	0.03399	0.51157
Erdös-Rényi (ER)	0.033	7.534	0.002956	0.78902

Simulation results for three different types of random graphs with 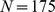 nodes, averaged over 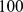 runs. “Pre” and “Post” stand for before and after circuit inductances are changed to reduce the gap between the graph Laplacian's first two eigenvalues. Note that *pre* and *post* circuits have the same graph topology and differ only in their inductances.

Note that we have made available open-source Python implementations of all algorithms described in this work. The Python code, together with R code used for plotting, has been posted on a public repository. This enables the reader to reproduce all results in this paper. Instructions on how to download this code is given below.

### Connections to Other Systems

We can make several connections between the problem studied in this paper and other problems of interest:

- **Random elastic networks.** Using a mechanical analogy between inductorscapacitors and massessprings, the nonlinear electronic network can be transformed into a mathematically equivalent network of masses and anharmonic springs [19, Appendix I]. Such random elastic networks have been of recent interest as models of amorphous solids [20]–[22]. For such networks, quartic spring potential energies have been considered [23]. Nonlinear random elastic networks have also been used to model molecular machines; in this context, tuning the gap between the first two eigenvalues of the linearized system enables the construction of networks with properties similar to those of real proteins [24]. Despite this activity, algorithms for computing and manipulating the frequency response of nonlinear elastic networks have not been developed. Our work addresses this issue directly.
- **Nonlinear electromagnetic media.** The circuit we analyze, for particular values of the circuit parameters, arises naturally as a finite volume discretization of Maxwell's equations for TE/TM modes in a nonlinear medium [25], [26]. The arbitrary connected graph topology of the circuit corresponds to a finite volume discretization on an arbitrary unstructured mesh. The algorithms developed here can be used to compute and optimize the frequency response of nonlinear electromagnetic media.
- **Coupled phase oscillator networks.** There has been intense interest in nonlinear phase oscillator networks, primarily due to the ability of such networks to model biophysical systems featuring synchronization. Though synchronization is not of primary interest in our system, we may still draw parallels. The effect of network topology on the properties of coupled phase oscillators has been studied extensively [27]–[30]. Manipulating eigenvalues of the Laplacian matrix enables one to enhance a network's synchronization properties [31]. More recently, several authors have developed algorithms for optimizing the synchronization of phase oscillator networks [32]–[37]. The questions considered in this subset of the coupled phase oscillator literature are related to the issues addressed in the present work.

## Methods

### Problem Formulation

Let 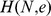 be a connected, simple graph with 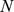 nodes and 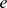 edges. Each edge corresponds to an inductor that physically connects two nodes. Each node corresponds to a capacitor and resistor, wired in parallel, that physically connect the node to a common ground. Let 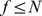 be the number of nodes that are driven by prescribed sources. Since the voltage at the prescribed source is known, we do not model it using a node. The connection between the source and the node that it drives is modeled by a half-edge, also known as a dangling edge since one end is connected to a driven node and the other end does not connect to any node. We let 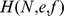 denote the graph together with the 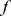 half-edges.

The capacitance and conductance (inverse resistance) at node 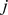 are 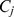 and 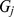, respectively. We let 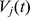 denote the voltage from node 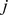 to ground at time 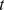. The inductance of edge 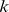 is 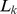, while the current through edge 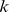 at time 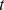 is 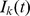. The exact dimensions for each component of 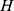, along with the currents and voltages, are tabulated in Table 2.

**Table 2:** Summary of the notation used in the paper.

Notation	Significance	Size
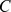	Capacitance at node	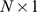
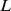	Inductance of edge	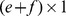
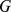	Conductance at node	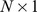
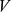	Voltage at node	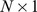
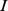	Current through edge	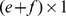
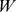	Input forcing	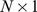
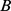	Signed incidence matrix	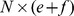

In order to write down Kirchhoff's laws, we must choose an orientation of the edges. The orientation of an edge records the direction of positive current flow through the edge. If we solve the problem with opposite orientations, the only difference we will notice is that the currents will pick up a factor of 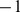. Consequently, the orientation we choose does not affect the solution in any material way. In what follows, we will choose a random orientation of the edges.

**Figure 4:**
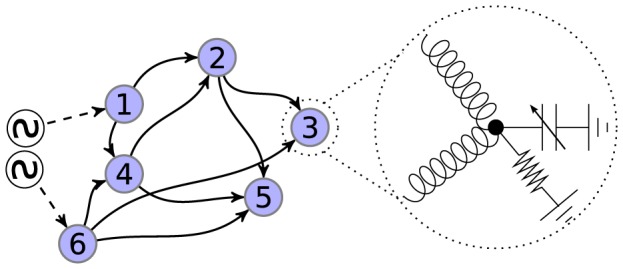
An example of a nonlinear electrical network. In the graph on the left, the numbered circles are nodes, the solid arrows are edges, and the dashed arrows are half-edges. Orientation of the arrows indicates the direction of positive current flow. Each node corresponds to a voltage-dependent capacitor to ground, wired in parallel with a resistor to ground, as depicted in the zoomed-in schematic for node 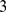. Each edge corresponds to an inductor. Each half-edge connects one prescribed voltage source to one given node. In this paper, all methods that are developed are valid for connected graphs with at least one half-edge. Note that the circuits in Figures 1–3 can all be represented using this graph formalism.

In Figure 4 we show an example graph corresponding to 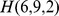. The edges are oriented randomly. The inputs are connected at nodes 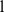 and 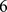 through two inductors. These input nodes correspond to half-edges in 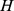. On the right we view node 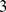 in detail. Each of the two edges connected to this node correspond to an inductor. A capacitor with capacitance 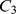 and a resistor with conductance 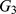 connect node 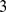 to ground.

To arrange Kirchhoff's laws compactly, we use the 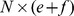 incidence matrix of 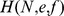, denoted by 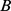. Let 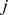 be an edge connecting the nodes 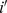 and 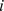. If 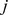 is oriented such that positive current starts at node 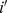 and flows to node 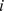, we write 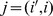. If 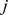 is a half-edge attached to node 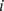, we write 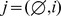, leaving the first slot empty and orienting the half-edge so it always points toward the forced node. The entries of the incidence matrix 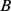 are
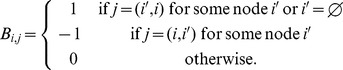



This paper will only consider single frequency time-harmonic forcing of the form 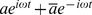 where 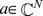. Let 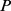 be an 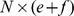 matrix with entries 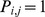 if node 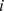 is connected to an input edge 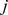 and 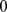 otherwise. Using the projection matrix 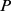 we define the forcing
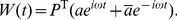
(1)


Using the notation summarized in Table 2, Kirchhoff's laws for the nonlinear circuit on the graph 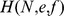 can now be written compactly as
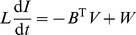
(2)

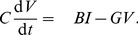
(3)


Here 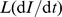, 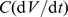, and 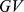 are examples of component-wise multiplication of vectors. For 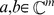, we define 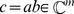 by 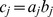 for 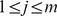. Note that in this case, we can also write 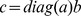. Here 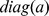 is the 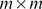 matrix that contains the vector 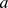 along its diagonal (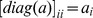) and is zero elsewhere.

The formulation (2–3) generalizes previous formulations [25], [38] in which the capacitors were constant and the systems considered were linear.

By differentiating (3) and inserting it into (2), we obtain a second-order system for the voltages:
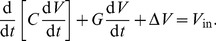
(4)


Here
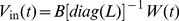
(5)

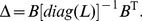
(6)


Note that 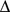 is the weighted Laplacian for the network with edge weights given by reciprocal inductance.

We assume that the capacitance at node 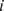 depends on the voltage at node 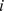:
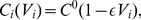
(7)where 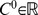 is a constant. Note that this choice of capacitance function means that (4) features a quadratic nonlinearity.

We can then formulate the *frequency response problem* for the nonlinear electrical network: given the amplitude vector 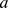 and frequency 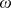 for the forcing function (1), determine the steady-state solution 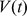 of (4).

### Perturbative Algorithm

We first solve the frequency response problem using a perturbative expansion in powers of 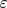. We use dots to denote differentiation with respect to time. Substituting the capacitance function (7) in (4) and rearranging, we obtain
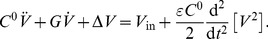
(8)


We expand
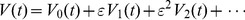
(9)


Inserting (9) into (8), we obtain equations for each order of 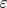. At zeroth order, we obtain
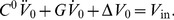
(10)


For 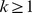, the 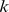-th order equation is
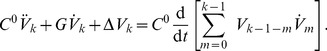
(11)


We now solve (10–11). Let us introduce the Fourier transform in time,
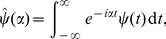
(12)with inverse Fourier transform



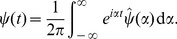
(13)Note that with these definitions,
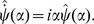



This implies that the Fourier transforms of both sides of (10–11) can be summarized by writing
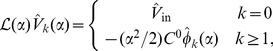
(14)where 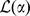 is the linear operator
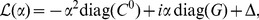
(15)and



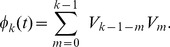
(16)By (5) and (1), we see that
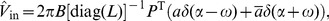
(17)where 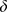 is the Dirac delta. Then the 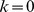 branch of (14) yields



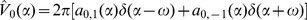
(18a)

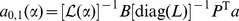
(18b)

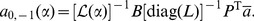
(18c)


Using the inverse Fourier transform, we have
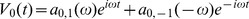


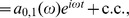
where “c.c.” stands for the complex conjugate of the previous terms. Here we have used the property that 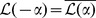.

Once we have computed 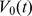, we can insert it into (16) to compute 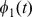. We will find that 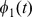 is a linear combination of 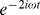, 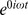, and 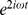. Using this fact in the 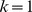 branch of (14), we can solve for 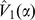 and then apply the inverse Fourier transform to compute 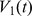. We will find that 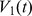 contains the same modes as 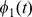.

The above shows how we get the perturbative solution algorithm started. Now let us move to the more general case where we seek 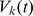 for any 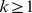. Assume that we have already computed 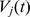 for 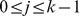, and that the solution takes the following form:
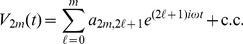
(19a)

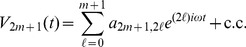
(19b)


In words, 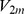 contains odd modes 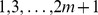, and 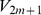 contains even modes 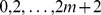. Here we assume that 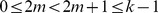, and that the 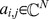 coefficients are known.

In order to solve for 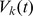, we use the 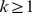 branch of (14), which requires us to compute (16). We have two cases, when 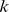 is odd and when 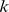 is even. In both cases, it is a simple (if tedious) algebraic exercise to show that 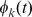 yields:

- when 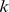 is odd, a sum of even Fourier modes 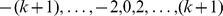, and
- when 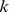 is even, a sum of odd Fourier modes 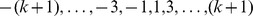.

In both cases, it is clear that using (14) to solve for 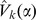 results in a sum of Dirac delta's. Applying the inverse Fourier transform yields 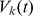, which will be a sum of Fourier modes. One can check that 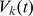 will have precisely the form (19a) or (19b) depending on whether 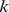 is even or odd, respectively.

The algorithm is then clear. Starting with (19), we apply component-wise multiplication to particular pairs of the 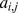 vectors in order to compute the coefficients of the Fourier modes of 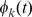 defined in (16). Next, we combine the step of solving for 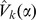 using the 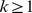 branch of (14) together with the step of computing the inverse Fourier transform. After component-wise multiplication of the Fourier coefficients of 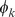 by 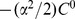, we multiply each coefficient on the left by 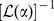 with 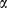 set to match the frequency of the corresponding Fourier mode. Dividing these coefficients by 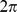 yields the Fourier coefficients of 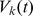, as desired.

While we have presented the algorithm in an intuitive way, the statements made above can be made rigorous, and a convergence theory for the perturbative expansion (9) can be established. This is the subject of ongoing work.

There are a few brief remarks to make about the algorithm presented above:

- As described above, we consider only those networks that contain resistance at all nodes, i.e., 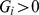 for all nodes 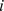. Such an assumption is not only physically realistic; it also guarantees that for all 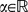, the matrix 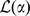 is invertible. The invertibility for the 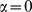 case is a consequence of Corollary 1 proved below.
- In this work, we are interested in the weakly nonlinear regime where 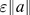 is sufficiently small such that the perturbative method converges. As the nondimensional constant 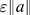 is increased beyond the breakdown point of the perturbative method, direct numerical solutions of the equations of motion reveal subharmonic oscillations, and eventually, chaotic oscillations.
- The fact that the Fourier transform yields the steady-state solution has been explained in our earlier work [7]. By fixing an arbitrary set of initial conditions and using the Laplace transform to derive the full solution, one can show that after the decay of transients, the part of the solution that remains is precisely what we obtain using the Fourier transform. This also explains why it was not necessary for us to specify initial conditions for (4) in our derivation above—the initial conditions only influence the decaying transient part of the solution.

### Iterative Algorithm

The perturbative method developed above shows us that the solution 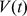 is a sum of harmonics where the fundamental frequency is given by the input frequency 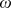. This implies that the steady-state solution 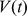 is periodic with period 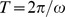. This observation leads us to ask whether it is possible to directly solve for the Fourier coefficients of 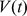 without first expanding in powers of 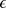. In this section, we develop a fixed point iteration scheme that accomplishes this task.

First, we integrate both sides of (8) from 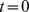 to 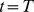 to derive
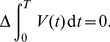
(20)


We show below that as long as the network contains at least one half-edge, 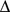 is invertible. Hence (20) implies
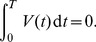
(21)


This means there is no zero/DC mode present in 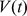, motivating the Fourier series expansion
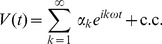
(22)


In order to compute the solution, we truncate at 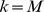, leading to an approximation 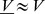:
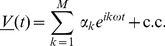
(23)


Using orthogonality we derive
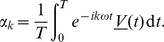



Using the 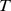-periodicity of 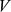 and integration by parts, we have
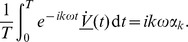



To simplify notation, we combine (1) and (5) and write 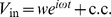 where
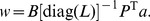
(24)


Now let 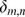 denote the Kronecker delta function which equals 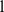 if 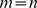, and 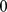 otherwise. We multiply both sides of (8) by 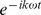, integrate from 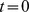 to 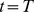, and finally divide by 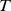 to obtain
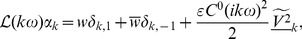
(25)where 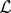 was defined in (15) and



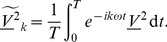
(26)Because the form of the nonlinearity is simple, we can insert (23) into (26) and derive
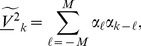
(27)with the understanding that 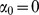, 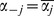 for 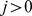, and 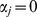 for 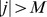. We insert (27) into (25) and obtain



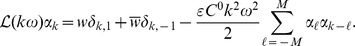



We convert this into an iterative scheme in a natural way. Let 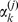 denote the 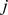-th iterate, and assume that 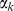 terms appearing on the left-hand side are at iteration 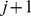, while those appearing on the right-hand side are at iteration 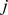. Let 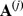 denote the 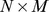 complex matrix whose 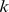-th column is 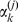. Then the scheme is
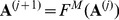
(28)where the 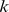-th column of the matrix 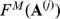 is



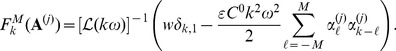
(29)Here we assume 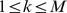, which is also why we have deleted the second Kronecker delta from the right-hand side.

Starting at 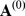, we iterate forward using (28), stopping the computation when 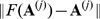 is below a specified tolerance. Note that in our implementation of 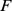, we precompute and store the LU factorization for the 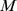 matrices 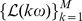, since this part of the computation of the right-hand side of (29) does not change from one iteration to the next.

Again, we have derived the algorithm but have not proven its convergence. Instead, we will demonstrate empirically that the algorithm converges using several numerical tests.

### Inverse Problem

In this section, we consider the inverse problem of finding a set of inductances such that 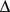, the Laplacian defined by (6), achieves a desired spectrum. Before describing an algorithm to solve this inverse problem, we review basic spectral properties of 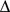.

**Lemma 1.** *Assume all inductances are positive. Then 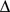 as defined in (6) is symmetric positive semidefinite, and all its eigenvalues must be nonnegative.*

*Proof.* Let 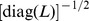 be the diagonal matrix whose 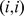-th element on the diagonal is 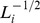, for 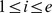. Since 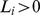, the matrix 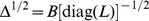 is real. Then 
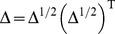
, and for any 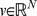, we have 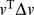  =  
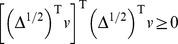
.

Let 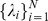 denote the spectrum of 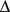, with eigenvalues arranged in nondecreasing order: 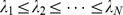. The above argument shows that 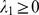. We can be more precise about this: if there are no half-edges, then 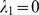, while the presence of at least one half-edge causes 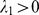.

**Lemma 2.** *Let 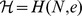 be a connected graph with 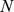 nodes, 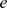 edges, and zero half-edges. For a particular orientation of the graph, let 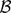 denote the signed incidence matrix. Then* 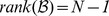.

*Proof.* Let 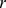 be any integer from 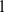 to 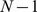. Consider any subset 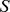 of 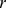 vertices of the graph. Take the sum of the rows of the incidence matrix corresponding to the elements of 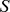. This sum cannot be zero; if it were, there would be no path connecting 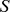 to the complement 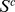 and the resulting graph would not be connected. Hence the sum of these rows must contain a nonzero entry. As the same would be true if we considered linear combinations of the rows corresponding to 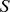, we conclude that any subset of at most 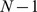 rows must be linearly independent. At the same time, if we take the sum of all the rows we get a zero row, because each column contains precisely one 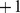 and one 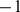.

**Lemma 3.** *Let 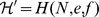 be a connected graph with 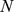 nodes, 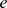 edges, and 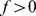 half-edges. For a particular orientation of the graph, let 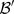 denote the signed incidence matrix. Then 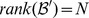.*

*Proof.* Without loss of generality, we can assume that the 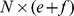 incidence matrix 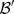 is organized such that the first 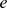 columns correspond to full edges, while columns 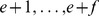 correspond to half-edges. Now choose any 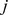 such that 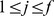, and examine column 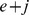 of 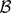. Let 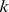 be the unique row in which this column contains 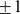. Since row 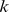 of 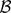 is the only row that contains an entry in column 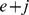, row 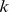 is linearly independent from the other 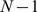 rows of 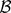. By Lemma 2, the submatrix of 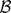 consisting of all rows other than row 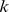 has rank 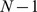. Including row 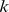 increases the rank by one, yielding a rank 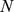 matrix.

**Lemma 4.** *For a connected graph 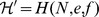 with 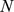 nodes, 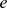 edges, and 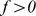 half-edges, let 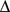 be the edge-weighted graph Laplacian defined in (6). Assume all inductances are positive. Then 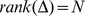.*

*Proof.* The 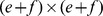 diagonal matrix 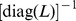 has rank 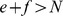. Let 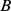 be the signed incidence matrix for a particular orientation of 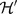. By Lemma 3, 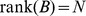, implying 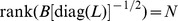, which implies 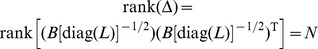.

**Corollary 1.** *Let 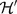, 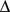 and the inductances satisfy the hypotheses of Lemma 4. Then 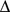 is symmetric positive definite and all eigenvalues of 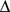 are positive, i.e., 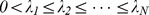.*

*Proof.* Combine Lemmas 1 and 4.

We now describe an algorithm that quantifies how we must change the vector of inductances 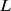 in order to make 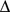 have a desired set of eigenvalues. In what follows, we assume we work with a system that satisfies the hypotheses of Corollary 1.

For 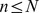, let 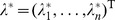 denote a vector of desired eigenvalues satisfying
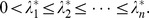



We treat the vector of inductances 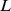 as a variable, and let 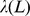 denote the sorted vector of eigenvalues of the graph Laplacian 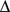 defined in (6). Since 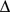 is symmetric, it possesses an orthonormal basis of eigenvectors. We assume that 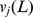 is the normalized eigenvector corresponding to 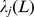.

Now let 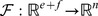 be the function
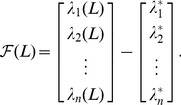
(30)


We now apply a version of Newton's method to find a zero of this function. To use Newton's method we will need to compute the Jacobian 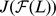. Let primes denote differentiation with respect to 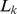. To form the Jacobian we need to find
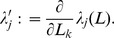



We proceed by implicit differentiation, starting from the eigenvector equation
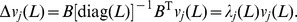



Differentiating both sides with respect to 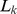, and omitting the dependence on 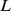, we obtain
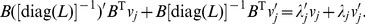
(31)


Since 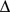 is symmetric,
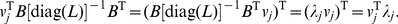
(32)


Multiplying (34) on the left by 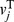 and using (35) together with 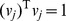, we obtain
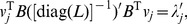
(33)where



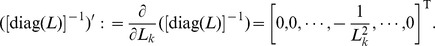



Using 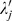 we can compute the entries of the Jacobian matrix and the corresponding Newton's method with pseudoinverse becomes
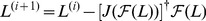
(34)where 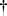 denotes the Moore-Penrose pseudoinverse.

Using (34) as shown might produce inductances such that the ratio of the largest to smallest inductance is too large. In order to avoid these large variations, we constrain 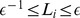. We incorporate these constraints using an active set approach, replacing 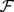 by the function 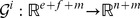, where 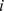 denotes the iteration number and 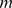 denotes the number of constraints violated by 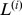. Let 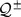 denote the functions
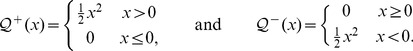
(35)


For every constraint 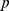 violated from below, we set 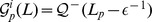. For every constraint 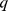 violated from above, we set 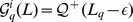. Since the 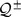 functions are continuously differentiable, it is easy to compute the Jacobian 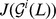 and then apply the algorithm
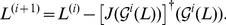
(36)


Algorithm (36) can be used to alter all the eigenvalues of the system if 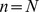 and 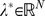. Alternatively, one can set 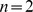 and only request the two smallest eigenvalues to be changed to 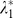 and 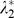, respectively.

In the next section we show that altering the lowest eigenvalue 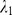 is enough to cause higher energy transfer to the higher modes. To show, we will use (39) to change 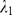 to some desired value, keeping 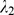 constant. We note that since we do not constrain 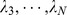, they can change as a result of altering 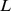, but 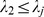 for 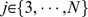 will be maintained.

For all applications of this inverse problem algorithm described in the next section, we use (36) with the initial conditions 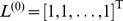 and the constraint violation parameter 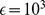.

### Gap Tuning: Methodology

How does the steady-state voltage in the nonlinear circuit change as a function of the gap between the first two eigenvalues of the graph Laplacian 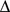
? In this section, we address this question by combining the perturbative/iterative algorithms with the inverse problem algorithm. We describe numerical experiments designed to test the effect of closing the graph Laplacian's first eigenvalue gap on the circuit's ability to (a) transfer more energy to higher harmonics, and (b) generate higher-amplitude output signals.

We conduct our numerical experiments on three types of random graphs, all generated using the NetworkX package [39]:

- Barabási-Albert (BA), a preferential attachment model with one parameter, 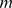, the number of edges to draw between each new node and existing nodes [40]. We set 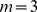 in our experiments.
- Watts-Strogatz (WS), a small world model with two parameters, 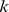, the number of nearest neighbor nodes to which each node is initially connected, and 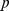, the probability of rewiring each edge [41]. In our experiments, we set 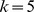 and 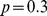.
- Erdös-Rényi (ER), a classical model in which edges are drawn independently with uniform probability 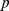 [42]. In our experiments, we set 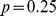.

When we produce realizations of any of these graphs, we accept only those graphs that are connected. Suppose we have used one of these three models to generate a connected, random graph with 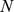 nodes. To make this a concrete circuit problem, we set 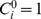 for all nodes 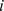, and 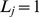 for all edges 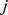. We fix the nonlinearity parameter 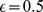. We select 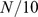 nodes uniformly at random, and attach half-edges to these nodes with inductance 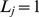. For each node 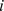, we set the conductance 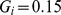 for the BA and WS graphs, and 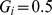 for the ER graphs. This selection will be explained in more detail below.

With these parameters set, we have enough information to compute the graph Laplacian 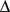 defined by (6). As we did before, let 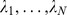 denote the eigenvalues of 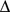 sorted in increasing order. We set the forcing frequency 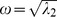. Since this value is a resonant frequency of the linear, undamped system we expect it lies close to a resonance for the nonlinear, damped system. The type of forcing we consider is 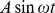, a special case of (1) with 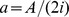.

With this setup, we use both the perturbative method and the iterative method to compute the steady-state solution 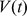. For the perturbative method, we solve up to order 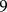, and for the iterative method, we solve using 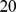 modes. This means that the iterative scheme captures ten modes—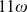 through 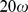
—that are not captured by the perturbative scheme. We compare the two solutions as a check for whether the number of modes we have considered is sufficient. In all tests, we find that there is no significant difference between the solutions, implying that the first 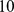 harmonics—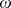 through 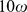
—are sufficient to resolve the solution.

Since 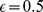, the capacitance model (7) is valid only for 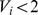. For all computed solutions, we check that the maximum voltage across all nodes in one cycle satisfies this constraint.

One quantity of interest in our simulations is the amount of energy in the higher harmonics. Let 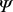 be an 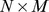 complex matrix such that the 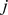-th column of 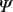 contains the Fourier coefficients of the 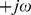 mode over all 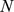 nodes. Here 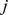 goes from 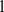 to 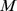, the maximum mode to which the solution is computed. We then define
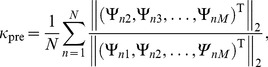
(37)the fraction of energy in modes 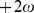 and higher, averaged over all nodes. We also compute
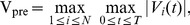
(38)the maximum magnitude voltage produced anywhere in the circuit during one period. For both 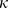 and 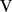, the subscript “pre” denotes that these quantities have been computed before we change 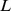 to manipulate the eigenvalues of 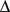.

Having computed 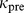, we now study how this fraction changes when we reduce the gap between the first two eigenvalues of 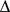. For a fixed 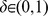, we set 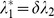 and 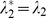, and then apply the inverse problem algorithm.

Using (36), we solve for a vector of inductances 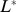 such that the first two eigenvalues of 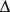 are given by 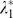 and 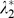. When we iterate forward using (36), if we find that 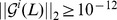 after 200 iterations, we generate a new random graph and restart the experiment.

We recompute the graph Laplacian 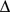 using the new vector inductances 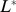, and again apply the perturbative and iterative algorithms to solve for the steady-state solution 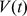. Using this solution, we compute the energy in the higher harmonics using the right-hand side of (37), now labeling this average fraction as 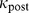. We also compute the right-hand side of (38) and label this quantity as 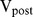.

Let us now describe how we choose the particular values of the conductance 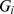 and the eigenvalue fraction 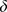. In Table 3, we tabulate 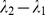, the gap between the second and first eigenvalue for each of the three types of random graphs described above. The eigenvalue gaps we present are averaged over 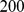 simulations for each of four graph sizes: 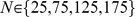.

**Table 3:** Eigenvalue gaps for random graphs.

	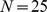	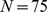	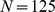	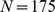
Barabási-Albert (BA)	0.6408	0.3561	0.3155	0.2850
Watts-Strogatz (WS)	1.4180	1.3255	1.2970	1.2758
Erdös-Rényi (ER)	1.5469	8.6936	17.7061	26.5297

For each of three types of random graphs, we vary the number of nodes 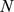 and record the first eigenvalue gap 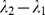. The displayed results have been averaged over 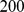 realizations.

We observe that the eigenvalue gaps for the BA and WS graphs do not change appreciably as a function of 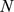, while for ER graphs, the gaps grow quickly as a function of 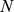. Our choice of 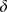 is guided by these results. For BA and WS graphs, we choose 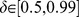. For ER graphs, we choose 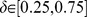.

When we solve for the steady-state voltages on these three types of graphs, we also notice a difference. For ER graphs, the maximum voltage grows quickly as a function of 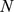, while for BA and WS graphs, the same phenomenon does not occur. To counteract the large growth of maximum voltages for large graph sizes, we set the conductance 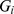 to the larger value of 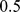 for ER graphs, causing more energy to dissipate through resistors. For BA and WS graphs, we set 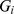 to 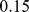.

## Results and Discussion

### Comparison of Steady-State Algorithms

In this section, we compare steady-state solutions computed by numerical integration against the solutions computed using the perturbative and iterative methods derived earlier.

For the tests described in this section, the domain is a 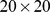 square lattice with 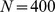 nodes. Nodes along the left and bottom boundaries of the lattice are driven by input forcing. The input provided is 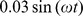 with 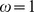. For the capacitance model (7), we set 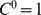 and 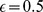. For each edge 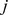, we set 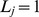. The conductance 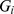 is set to 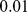 at all points except for the top-right corner, where it is set to 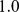.

To compare the results of the perturbative and iterative methods against the numerical integrator, we will need to obtain the steady-state solution using the numerical integrator. To do this, we start at 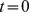 and numerically integrate the first-order system (2–3) forward in time for 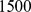 cycles. The ODE solver uses the Dormand-Prince (*dopri5*) method with relative and absolute tolerances equal to 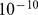 and 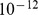, respectively. For the parameters given above, this number of cycles is sufficient so that, from one cycle to the next, the change in the solution is on the order of the relative tolerance of the numerical integrator. Hence we take the solution over the last cycle to be the steady-state solution.

As a preliminary check, we directly compare the three steady-state solutions. We treat the solution obtained from numerical time integration as a reference solution 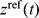. Let 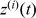 denote either the perturbative or iterative solution after 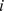 iterations—for the perturbative method, the iteration count is defined as the largest mode number present in the solution. Let 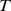 be the period of the steady-state solution, and for an integer 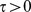, consider the equispaced discretization of the interval 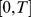 given by 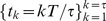. For each iteration 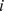, we evaluate both the the perturbative/iterative and reference solution on this equispaced grid with 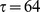 points, and we compute the error
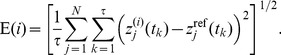
(39)


**Figure 5:**
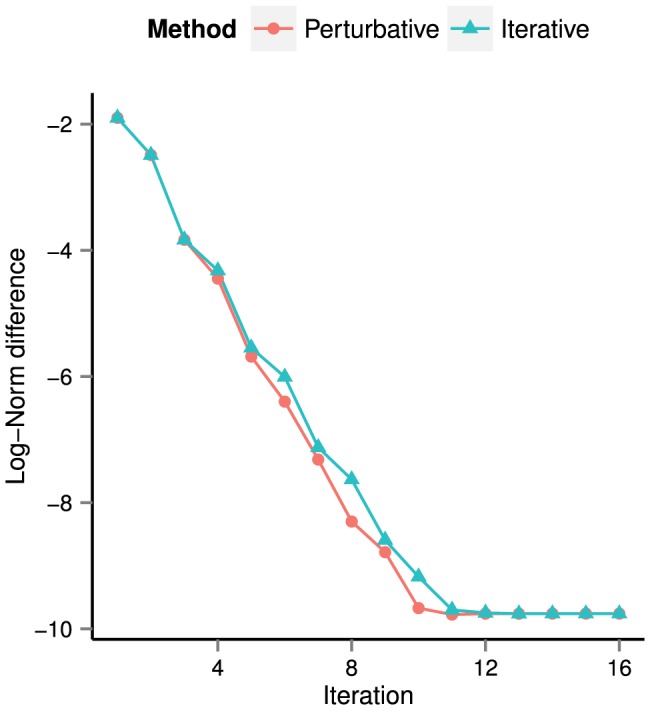
Error between perturbative/iterative solutions and reference solution. The reference solution has been computed via numerical time integration. We plot the log of the error as a function of the number of iterations. As shown in Figures 6 and 7 together with Tables 4 and 5, the perturbative/iterative solutions are more accurate than the reference solution. This explains why, in the above plot, the perturbative and iterative solutions do not converge to the reference solution.

In Figure 5 we have plotted 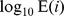 as a function of the iteration 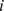. While both methods initially tend towards the reference solution, we see from Figure 5 that the error does not drop below 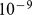. In the following subsections, we provide evidence that the reference solution is less accurate than the solutions computed using the perturbative/iterative methods. This explains why the error in Figure 5 does not go to zero, i.e., why the perturbative/iterative methods will not converge to the solution produced by time integration.

Our first tests concern the Fourier coefficients of the computed solutions. In what follows, we use 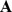 to denote the vector of Fourier series coefficients associated with a steady-state solution computed using any of the three methods discussed above.

#### Fixed Point Error

Suppose that 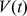 is an exact 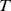-periodic steady-state solution of (4). If we were to expand this solution in a Fourier series as in (22), the resulting (infinite) coefficient vector 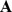 would satisfy 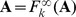 for all 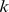, with 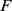 as in (29).

In both the perturbative and iterative methods, what we seek is a finite-mode truncation of the exact solution. For the iterative method we fix 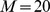 so that the highest mode has frequency 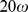. In the perturbative method we solve, we solve up to order 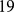, which implies that the highest mode in the solution has frequency 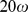.

Combining the ideas of the last two paragraphs, it is natural to use
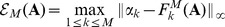
(40)as an error metric for the 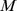-mode truncation of the exact solution. In Table 4, we record (40) for solutions computed using the perturbative, iterative, and numerical integration methods. Note that the iterative and perturbative methods directly provide us with the Fourier coefficients necessary for this calculation. We compute the Fourier coefficients of the numerical integrator's solution using the FFT. Table 4 shows that the perturbative and iterative solutions are about five orders of magnitude closer to being fixed points of 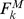 than the solution obtained from numerical integration.

**Table 4:** Comparison of the three solutions using the fixed point error metric (40).

Scheme	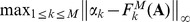
Numerical	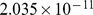
Perturbative	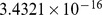
Iterative	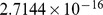

**Figure 6:**
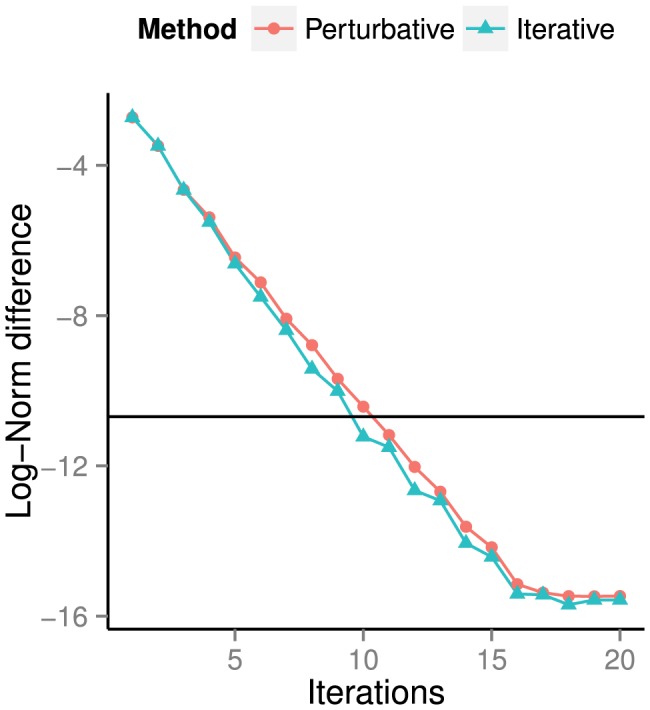
Log of the fixed point error (40) of the perturbative/iterative solutions after 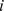 iterations. Up to iteration 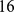, both curves are close to linear with slopes of 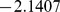 (perturbative) and 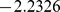 (iterative), indicating exponential convergence of both methods. Note that only 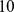 iterations are required to reach error values corresponding to that of the numerical integrator's solution.

For the perturbative and iterative methods, let us examine how the fixed point error (40) decreases as a function of iteration count. In Figure 6, we plot 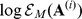 versus the iteration number 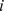. Here 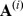 is the vector of Fourier coefficients for the solution computed after only 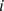 iterations. The plot shows that, for both the perturbative and iterative methods, approximately 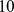 iterations are required to match the fixed point error of the solution computed using time integration. The error of this latter solution, taken from Table 4, is represented on Figure 6 by a horizontal black line.

Figure 6 also shows that the perturbative and iterative methods converge exponentially in the number of iterations. From iteration 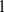 until iteration 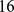, fitting lines of best fit to the perturbative and iterative error curves results in slopes of 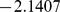 and 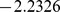, respectively. For both methods, this can be approximated by writing 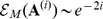. After 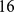 iterations, the error has approached machine epsilon, and both curves level off before reaching the final values shown in Table 4.

#### Decay Rate

Suppose we write the first-order system (2–3) in the form 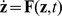, with 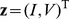. Then on the open set 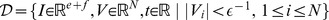, the vector field 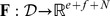 is 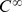, i.e., 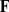 is 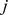 times continuously differentiable for any integer 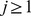. By the standard existence/uniqueness theorem for ordinary differential equations, it follows that wherever the solution 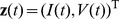 exists, it must also be 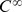 in 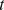.

The above observation enables us to test the decay of the Fourier coefficients of all three solutions. For if the steady-state solution 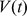 is 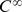 in 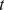, then the Fourier series coefficients of 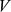 must satisfy the following decay property:
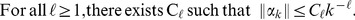
(41)


**Figure 7:**
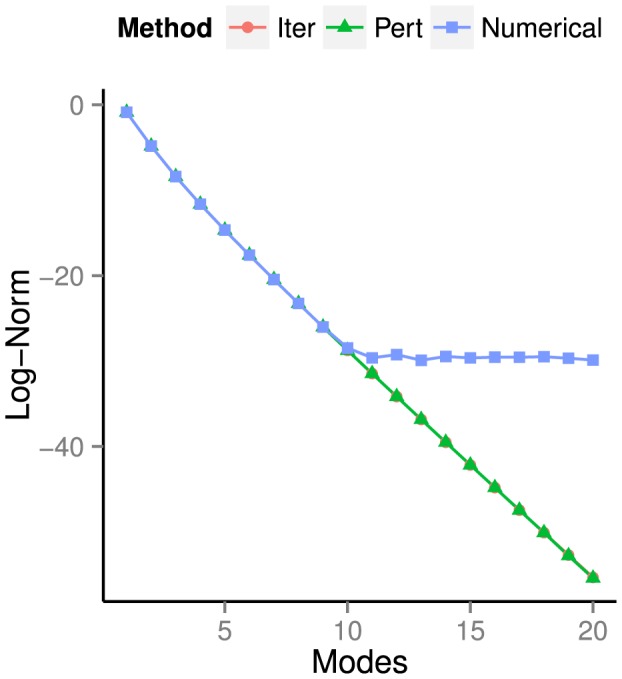
Decay of Fourier coefficients. We plot 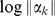 versus 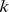 to illustrate the decay of Fourier coefficients for the three methods. The iterative and perturbative curves coincide and are nearly linear with slope 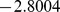; the exponential decay of these Fourier coefficients is consistent with theory. The time integrator's Fourier coefficients do not decay after mode 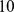, violating the theoretical decay rate.

To examine the decay of the Fourier coefficients for the three computed solutions, we plot 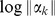 versus 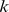 in Figure 7. For the perturbative and iterative solutions, the curves on the plot coincide and are nearly linear with slope 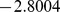. This implies that 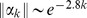, which is sufficient to satisfy the theoretical decay rate given by (41).

The Fourier coefficients obtained from the numerical integrator's solution, on the other hand, do not decay at all beyond mode 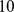. This violates the theoretical decay rate (41) even for 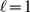.

#### Energy Conservation

Next we test the energy conservation properties of the three computed solutions. We proceed to derive an energy balance equation. Because our capacitors are voltage-dependent, the charge 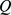 and voltage 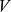 are related via 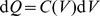, which implies
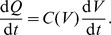



Using this in (3) together with (2), we obtain
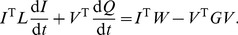
(42)


Let 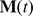 be the total energy stored in the magnetic fields of all inductors at time 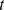. Then
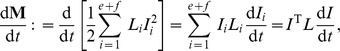
the first term on the left-hand side of (42). Let 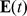 be the total energy stored in the electric fields of all capacitors at time 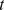. Then



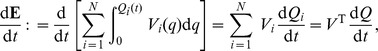
the second term on the left hand side of (42). Hence (42) reads:



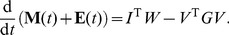
(43)If the system has reached a 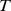-periodic steady state, then 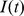, 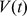, 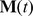, and 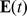 will all be 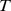-periodic. Therefore, integrating (43) in 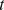 from 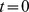 to 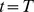, we find that the left-hand side vanishes. The remaining terms yield the following energy balance equation:
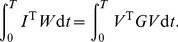
(44)


The left-hand side is the energy pumped into the system over one cycle, while the right-hand side denotes the energy dissipated through resistors, again over one cycle.

Table 5 shows the absolute difference between the left- and right-hand sides of (44), computed for each of the three methods. We find that for the perturbative and iterative methods' energy balance errors are below machine epsilon. The numerical integrator yields an error approximately five orders of magnitude larger than that of the two other methods.

**Table 5:** Comparison of the three solutions' preservation of the energy balance (44).

Scheme	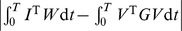
Numerical	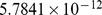
Perturbative	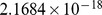
Iterative	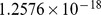

#### Computational Time

The results presented thus far indicate that whether we measure error using the fixed point error (40) or the violation of the energy balance (44), the solution obtained via numerical integration has errors that are approximately five orders of magnitude larger than that of the perturbative/iterative methods. The actual values of the errors committed by the numerical integrator in Tables 4 and 5, as well as the final error values for the curves in Figure 5, are close to the numerical integration relative and absolute tolerances of 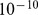 and 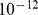, respectively. We hypothesize that, if computational time were not an issue, we could run the numerical integrator with smaller tolerances and obtain steady-state solutions that more closely match, in the same error metrics described above, the perturbative and iterative solutions.

As we now proceed to show, computational time is a major issue for the time integration method. In Table 6, we record the time required to compute steady-state solutions using the three methods. We see from the Time I column that to achieve the error of 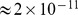 in Table 4, the numerical integrator requires over 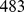 seconds. We know from Figure 6 that the perturbative/iterative methods require 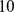 iterations to achieve approximately the same error as the time integrator; the remaining entries in the Time I column show that both the perturbative and iterative algorithms compute such a solution hundreds of times faster than the time integrator.

**Table 6:** Timing results for three frequency response algorithms.

Scheme	Time I (to achieve comparable error)	Time II (to achieve 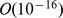 error)
Numerical	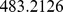 s	N/A
Perturbative	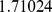 s	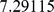 s
Iterative	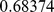 s	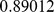 s

For the numerical method, Time I records the time required to integrate forward by 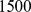 cycles and obtain a solution with fixed point error 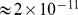 as in Table 4. For the perturbative/iterative methods, Time I records the time required to compute 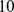 iterations, resulting in a fixed point error comparable to that of the numerical method—see the crossing of the curves with the black horizontal line in Figure 6. For the perturbative/iterative methods, we also record under Time II the time required to achieve the 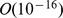 errors as in Table 4. All times are averaged over 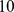 runs.

The Time II column in Table 6 records how long it takes the perturbative/iterative algorithms to achieve the errors recorded in Table 4. Observe that even if we run the perturbative/iterative algorithms all the way to full convergence, they are much faster than time integration. In this case, the time integrator is 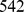 (respectively, 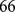) times slower than the iterative (respectively, perturbative) algorithm.

Note that the perturbative and iterative algorithms were implemented in Python using the Numpy/Scipy packages. The *dopri5* implementation used for numerical time integration is the implementation provided by the scipy.integrate.ode module. All times reported are average times across 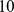 runs on the same machine.

### Gap Tuning

**Figure 8:**
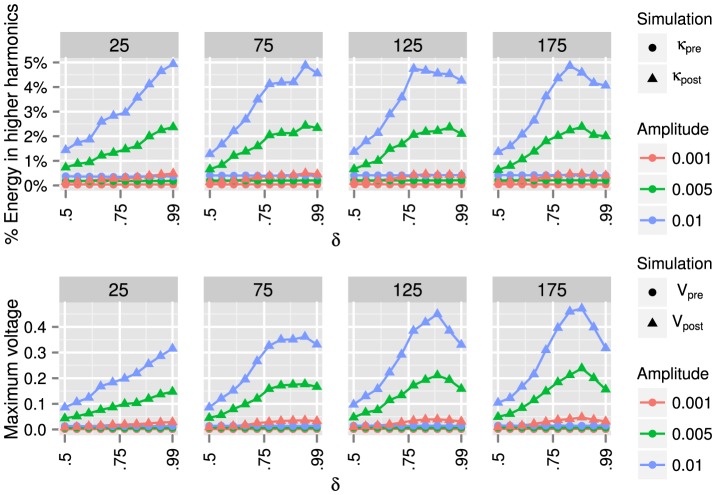
Barabási-Albert random graph results. From left to right, we present results for Barabási-Albert random graphs with 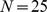, 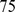, 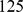, and 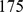 nodes. For each graph, we use algorithm (36) to modify the inductances 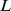 such that the ratio of the smallest to the second smallest eigenvalue is 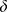. We use *pre* and *post* to denote, respectively, the graphs before and after algorithm (36) is applied. By shrinking the gap between the first two eigenvalues, the energy transferred to higher harmonics (37) can be increased from approximately 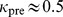
% to 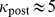
% (for all graph sizes), and the maximum voltage (38) can be increased from 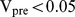 volts to 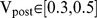 volts (depending on the graph size). We also note that for larger graphs, choosing 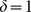 (i.e., no gap between the first two eigenvalues) does not yield optimal behavior.

**Figure 9:**
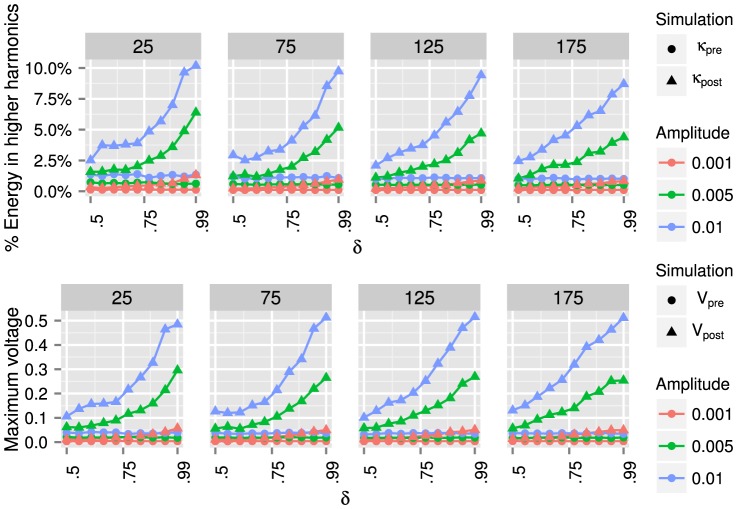
Watts-Strogatz random graph results. From left to right, we present results for Watts-Strogatz random graphs with 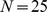, 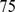, 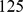, and 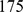 nodes. For each graph, we use algorithm (36) to modify the inductances 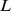 such that the ratio of the smallest to the second smallest eigenvalue is 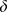. We use *pre* and *post* to denote, respectively, the graphs before and after algorithm (36) is applied. By shrinking the gap between the first two eigenvalues, the energy transferred to higher harmonics (37) can be increased from 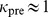
% to 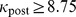
% (for all graph sizes), and the maximum voltage (38) can be increased from 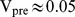 volts to 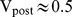 volts (for all graph sizes). The values of 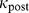 for Watts-Strogatz graphs are about twice as large as the values of 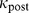 for Barabási-Albert graphs in Figure 8.

**Figure 10:**
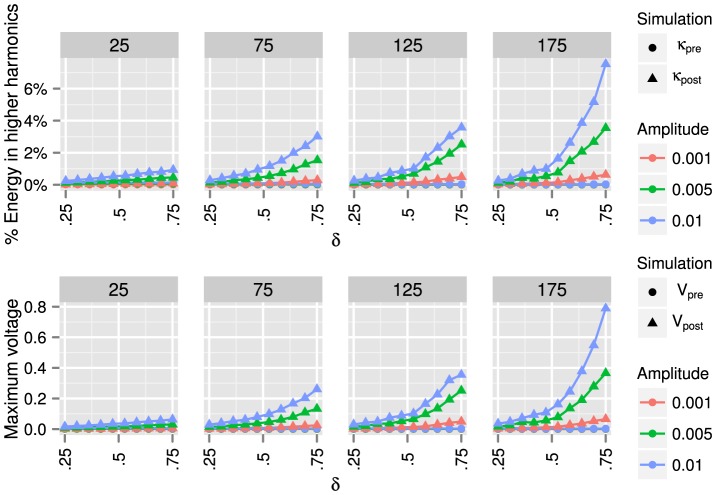
Erdös-Rényi random graph results. From left to right, we present results for Erdös-Rényi random graphs with 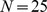, 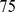, 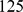, and 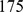 nodes. For each graph, we use algorithm (36) to modify the inductances 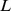 such that the ratio of the smallest to the second smallest eigenvalue is 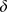. We use *pre* and *post* to denote, respectively, the graphs before and after algorithm (36) is applied. By shrinking the gap between the first two eigenvalues, the energy transferred to higher harmonics (37) can be increased to 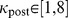
% (depending on the graph size), and the maximum voltage (38) can be increased to 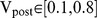 (depending on the graph size). The results for Erdös-Rényi graphs are much more strongly dependent on the number of nodes 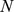 than the results shown in Figures 8 or 9. Note that the peak voltages for the 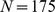 graphs with forcing amplitude 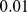 are the largest voltages for any graphs considered in this paper. We can increase the peak voltages for smaller graphs by choosing a smaller value of the conductance than 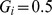 (for all nodes 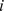), the value used to compute the results in this figure.

For each 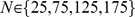, and for each of 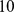 values 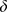 chosen in an equispaced fashion from the intervals given above, we compute 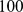 runs of the complete procedure described above—see Gap Tuning: Methodology. For each such run, we compute pre/post values of 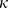 and 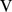 for three values of the input forcing amplitude, which we take to be the same at all input nodes 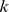: 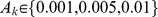. These results for 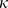 and 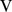, averaged over the 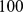 runs, are plotted in Figures 8, 9, and 10.

Figure 8 shows the results for Barabási-Albert (BA) graphs. By shrinking the gap between the first two eigenvalues, the percentage of energy transferred to higher harmonics (37) can be increased by approximately one order of magnitude, for all graph sizes, while the maximum magnitude voltage (38) can be increased by a factor of 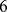 to 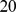, depending on the graph size. Note that for larger graphs, choosing 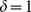, i.e., forcing the first two eigenvalues to coincide, does not yield optimal energy transfer to higher harmonics.

The results in Figure 9 for Watts-Strogatz (WS) graphs are similar to those for BA graphs. We again find that by shrinking the gap between the first two eigenvalues, the energy transferred to higher harmonics (37) can be increased. However, the values of 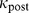 for Watts-Strogatz graphs are about twice as large as the values of 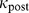 for Barabási-Albert graphs in Figure 8. For all graph sizes, tuning the eigenvalue gap can increase the percentage of energy transferred to higher harmonics by a factor of up to 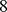, while the maximum magnitude voltage can be increased by approximately one order of magnitude.

In Figure 10, we present the results for Erdös-Rényi graphs. The results again support the finding that by shrinking the gap between the first two eigenvalues, the circuit can transfer more energy to higher harmonics and boost the peak magnitude of output signals. Specifically, we see that the energy transferred to higher harmonics (37) can be increased to 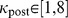
%, and the maximum voltage (38) can be increased to 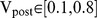.

The results for Erdös-Rényi graphs are much more strongly dependent on the number of nodes 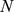 than the results shown in Figures 8 or 9. Note that the peak voltages for the 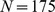 graphs with forcing amplitude 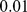 are the largest voltages for any graphs considered in this paper. We can increase the peak voltages for smaller graphs by choosing a smaller value of the conductance than 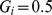 (for all nodes 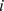), the value used to compute the results in Figure 10.

For all three types of graphs, both *pre* and *post* values of 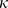 and 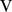 increase as a function of the input forcing amplitude.

### Code

All code necessary to reproduce the above results have been posted as a public repository on GitHub, accessible at the following URL: https://github.com/GarnetVaz/Nonlinear-electrical-oscillators

We use Python together with the numpy, scipy, matplotlib, and networkx modules for all numerical computing. The code that generates Figures 8, 9, and 10 is set to utilize 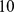 processors using the open-source multiprocessing module. For plotting, we use R together with the ggplot2, plyr, and reshape packages. All languages, packages and modules used are open source.

Assuming all packages and modules have been correctly installed, one can reproduce all results by running the Python codes numerical_comparison.py and graphmulti.py. The latter code may require several hours to run. The Python codes will generate figures using R; the R codes we provide need not be run independently.

Further details on how to run the codes, including the specific versions of required packages and modules, are given in the README.md file at the URL given above.

The code that we provide can easily be modified to run simulations not described here. For example, one can compare the perturbative/iterative algorithms against numerical integration using graphs other than the 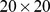 grid graph used above. One can also explore gap tuning results for random graphs with different parameters than the ones we have chosen.

## Conclusion

For nonlinear electrical circuits on arbitrary connected graphs, we have developed two numerical methods to compute the steady-state voltage. Using both absolute metrics and relative comparisons with a solution obtained via direct numerical integration, we validated the new algorithms. The results show that for the same error tolerance, both the perturbative and iterative methods are orders of magnitude faster than the solution obtained by time stepping. Moreover, these methods are able to capture the behavior in high Fourier modes and converge to machine precision in a fixed point error metric.

In future work, we plan to apply the steady-state algorithms developed above to solve Maxwell's equations in nonlinear electromagnetic media [26]. This application makes use of the correspondence between the nonlinear electrical network and a finite volume discretization of Maxwell's equations on an unstructured mesh.

In order to enhance the nonlinearity-driven features of these circuits, we developed a Newton-like algorithm that alters the eigenvalues of a network's graph Laplacian. The algorithm leaves the topology of the network untouched, changing only the inductances, i.e., the edge weights. By applying the Newton-like algorithm to three types of random graphs, we showed that reducing the gap between the graph Laplacian's first two eigenvalues leads to enhanced nonlinear behavior. Comparing pre- and post-optimized circuits, it is evident that optimizing the eigenvalue gap significantly increases (i) energy transfer from the fundamental driving frequency to higher harmonics, and (ii) the maximum magnitude output voltage.

In both the perturbative and iterative algorithm, the only way in which the network's structure influences its frequency response is through the graph Laplacian matrix. In our experiments, we have tuned this graph Laplacian's first eigenvalue gap by holding the topology of the graph constant and altering the edge weights, i.e., inductances. What if we had instead tuned the gap by holding the edge weights constant and altering the topology of the graph? This would amount to altering the incidence matrix 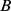 instead of the inductance vector 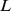. So long as both types of alterations result in the same graph Laplacian 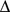, our results indicate that the nonlinear electrical network's functionality should be enhanced significantly. This, of course, leads to the question of whether it is possible to algorithmically alter the network topology to achieve a particular graph Laplacian matrix, an interesting avenue for further work.
